# Multiplex Detection of Fluorescent Chemokine Binding to CXC Chemokine Receptors by NanoBRET

**DOI:** 10.3390/ijms25095018

**Published:** 2024-05-04

**Authors:** Justyna M. Adamska, Spyridon Leftheriotis, Reggie Bosma, Henry F. Vischer, Rob Leurs

**Affiliations:** Amsterdam Institute of Molecular and Life Sciences, Division of Medicinal Chemistry, Faculty of Science, Vrije Universiteit Amsterdam, De Boelelaan 1083, 1081 HV Amsterdam, The Netherlandsspyleftheriotis@gmail.com (S.L.); h.f.vischer@vu.nl (H.F.V.)

**Keywords:** NanoBRET, multiplexing, chemokine receptors, GPCRs

## Abstract

NanoLuc-mediated bioluminescence resonance energy transfer (NanoBRET) has gained popularity for its ability to homogenously measure ligand binding to G protein-coupled receptors (GPCRs), including the subfamily of chemokine receptors. These receptors, such as ACKR3, CXCR4, CXCR3, play a crucial role in the regulation of the immune system, are associated with inflammatory diseases and cancer, and are seen as promising drug targets. The aim of this study was to optimize NanoBRET-based ligand binding to NLuc-ACKR3 and NLuc-CXCR4 using different fluorescently labeled chemokine CXCL12 analogs and their use in a multiplex NanoBRET binding assay of two chemokine receptors at the same time. The four fluorescent CXCL12 analogs (CXCL12-AZD488, -AZD546, -AZD594, -AZD647) showed high-affinity saturable binding to both NLuc-ACKR3 and NLuc-CXCR4, with relatively low levels of non-specific binding. Additionally, the binding of all AZDye-labeled CXCL12s to Nluc receptors was inhibited by pharmacologically relevant unlabeled chemokines and small molecules. The NanoBRET binding assay for CXCL10-AZD488 binding to Nluc-CXCR3 was also successfully established and successfully employed for the simultaneous measurement of the binding of unlabeled small molecules to NLuc-CXCR3 and NLuc-CXCR4. In conclusion, multiplexing the NanoBRET-based competition binding assay is a promising tool for testing unlabeled (small) molecules against multiple GPCRs simultaneously.

## 1. Introduction

Binding affinity is one of the key parameters in the identification and lead development of drug-like molecules for therapeutic targets and is often determined from competition binding curves against a target-specific labeled probe. Traditionally, radioactive ligands have been employed as labeled probes, but due to safety and cost concerns, fluorescent ligands are being developed as an alternative. Fluorescent target probes have been used in techniques such as the flow-cytometry-based binding assay or time-resolved fluorescence resonance energy transfer (trFRET) [1,2]. Additionally, the development of the bright and compact Nanoluciferase bioluminescent protein (NLuc; 19 kDa) has further increased the popularity of fluorescent ligands by measuring their target engagement via bioluminescence resonance energy transfer (BRET) [3,4].

In contrast to the classical radioactive binding endpoint assays that are terminated by the physical separation of a bound and unbound radioligand, both trFRET and NanoBRET-based binding assays are performed in a homogeneous format without separation steps, and ligand binding to a receptor can consequently be measured in real time [2,4]. Both trFRET and NanoBRET-based binding assays require the fusion of, respectively, the self-labeling protein SNAP-tag (19.4 kDa) or Nluc to the extracellular N-terminus of the receptor target of interest by DNA engineering and recombinant expression in cell lines [3,5,6]. The SNAP-tag is derived from the enzyme O6-alkylguanine-DNA-alkyltransferase and requires covalent pre-labeling with a Tb^3+−^cryptate fluorophore to function as a long-lifetime FRET donor allowing time-resolved detection [2]; whereas NLuc requires the addition of a substrate before measurement to act as a NanoBRET donor. This method has been successfully employed in studying ligand binding to G protein-coupled receptors (GPCRs), including the subfamily of chemokine receptors [7,8,9,10], which are activated by endogenous ligands called chemokines.

Chemokines are small (8–12 kDa) secreted proteins that signal through their corresponding receptors. Around 50 chemokines and their 24 receptors play a crucial role in the regulation of the immune system [11,12]. The dysregulation of the chemokines system has been linked to various pathological conditions such as inflammation, atherosclerosis and cancer [11,13]. Chemokine receptors can be categorized into two main groups: 19 classical chemokine receptors that signal via heterotrimeric G proteins (e.g., CXCR3 and CXCR4) and five atypical chemokine receptors (e.g., ACKR3, also known as CXCR7), which are G protein-independent and play crucial roles in scavenging chemokines from the extracellular environment through internalization and consequently regulating their availability for classical chemokine receptors [12]. CXCR4 interacts exclusively with CXCL12, while CXCR3 interacts with three different chemokines: CXCL9, CXCL10 and CXCL11. ACKR3’s activation and internalization are induced upon binding with CXCL11 and CXCL12. Hence, the availability of CXCL11 and CXCL12 chemokines in the micro-environment of CXCR3 and CXCR4, respectively, is regulated by their ACKR3-mediated scavenging [14,15].

Different ligand–receptor pairs (such as CXCL12-ACKR3/CXCR4) play roles in both pro- and anti-tumor functions in cancer, affecting processes like angiogenesis, proliferation, differentiation, and metastasis. Chemokine receptor overexpression varies among cancer types, such as breast and lung cancer [16,17]. Consequently, chemokine receptors are seen as promising drug targets. Small-molecule ligands targeting chemokine receptors such as ACKR3, CXCR4, and CXCR3 have been developed to explore the functioning of these GPCRs and to lay the groundwork for novel therapeutic strategies. For example, the FDA approved plerixafor/AMD3100 (CXCR4 antagonist) for hematopoietic stem cell mobilization. Additionally, this molecule has shown promising results in clinical trials for WHIM syndrome [18]. Furthermore, ACKR3 antagonist ACT-1004-1239 (VUF25550) [19] is currently in Phase 1 clinical trials, alongside other CXCR4 inhibitors such as burixafor (TG-0054) [20] and AMD070 (mavorixafor) [21], which are in Phase 3 clinical trials for breast cancer and WHIM syndrome, respectively. There are currently no FDA-approved drugs targeting CXCR3, and small molecules are currently not in clinical trials for this purpose.

**Figure 1:**
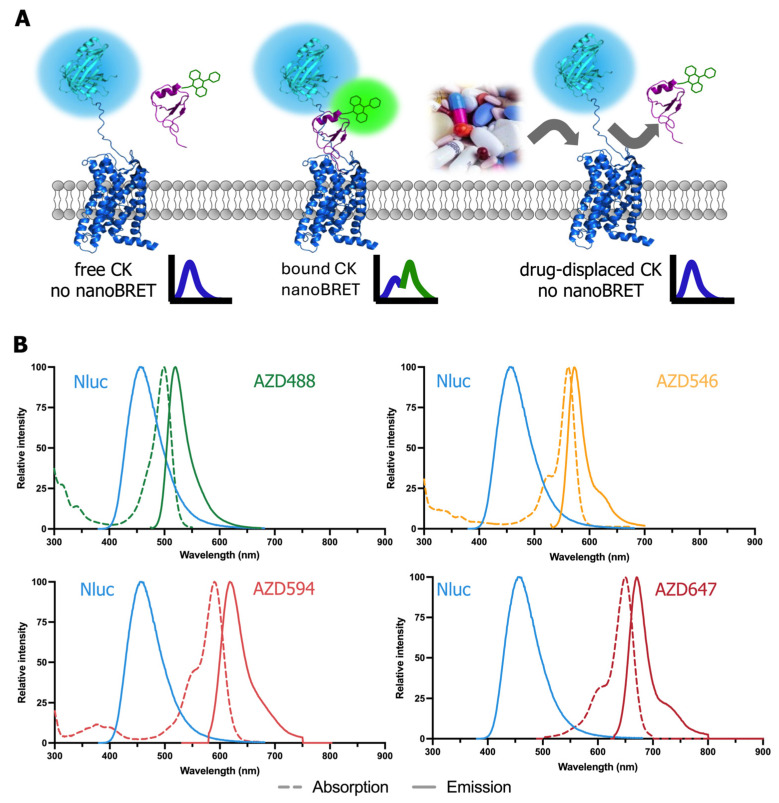
Principle of NanoBRET binding. (**A**) NLuc (light blue)-tagged receptors (dark blue) expressed on HEK293T membranes act as BRET donors and the chemokine (CK) (purple) labeled with AZDye (green) serves as the BRET acceptor. The binding of CK-AZDye to NLuc receptors increases the proximity between BRET partners, enabling BRET from donor to acceptor. The binding of unlabeled ligands to receptors inhibits CK-AZDye binding, resulting in no NanoBRET signal. (**B**) The emission spectrum of NLuc and the absorption (dotted line) and emission (solid line) spectra of the used AZDyes tagged chemokines as an acceptor in NanoBRET experiments.

Given the promising prospects of chemokine receptors as drug targets, our group has developed various small-molecule ligands such as the ACKR3 agonist VUF15485 [22], a CXCR3 inverse agonist VUF11211 [23], and CXCR4 antagonists [24]. However, there is still a need to develop/find new small molecules or other modalities to modulate their activity. Therefore, we developed and/or improved a NanoBRET-based screening methodology for the discovery of small molecules using several CXC chemokine receptors. This study characterized the binding of different fluorescent CXCL12 analogs to NLuc-fused ACKR3 and CXCR4 receptors (Figure 1A). Specifically, four fluorescent dyes with excitation maxima ranging from 494 nm to 649 nm that have been tagged to the C-terminus of CXCL12 by click chemistry were explored (Figure 1B). As shown in literature, NLuc is capable of supporting BRET to a wide spectral range of fluorescent acceptors [4,7]. Finally, the simultaneous use of different fluorescenly-tagged chemokines was explored to measure the interaction with their cognate receptor in a multiplex NanoBRET assay. This multiplexed NanoBRET-based binding assay allows the screening/testing of unlabeled (small) molecules against multiple GPCRs simultaneously.

## 2. Results

### 2.1. Fluorescent AZDye-Labeled CXCL12 Analogs Act as CXCR4 and ACKR3 Agonists

**Figure 2:**
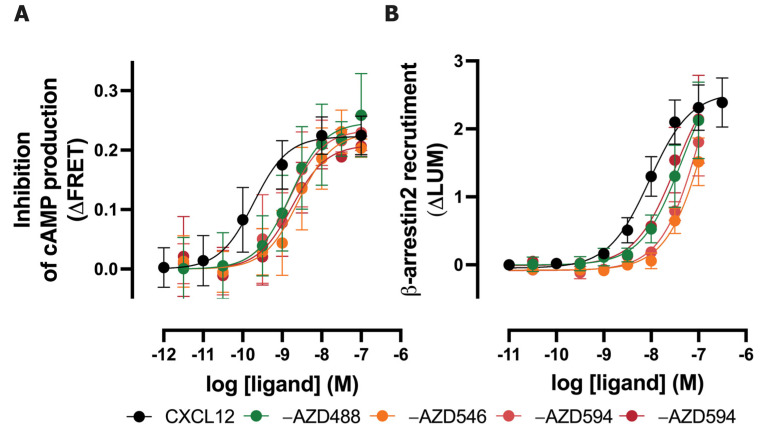
Effect of AZDye-labeled CXCL12 analogs on CXCR4 and ACKR3 activity. (**A**) Inhibition of isoprenaline-induced intracellular cAMP level in HEK293 cells stably expressing the FRET-based EPAC sensor and CXCR4 in response to CXCL12 and fluorescent CXCL12 analogs. (**B**) β-arrestin2 recruitment to ACKR3 in response to CXCL12 analogs was measured using a NanoBiT complementation assay in HEK293T cells. Data are shown as the agonist-induced FRET ratio (∆FRET) or luminescence (∆LUM) over vehicle, respectively. Data are shown as the mean ± SD of three experiments.

To evaluate whether the labeling of CXCL12 at the C-terminus with AZDyes via click chemistry affects the biological activity at its cognate receptors, the CXCR4-mediated inhibition of intracellular cAMP production and β-arrestin2 recruitment to ACKR3 were measured (Figure 2). Like CXCL12, the four AZDye-labeled CXCL12 analogs acted as full agonists on the inhibition of cAMP production in CXCR4-expressing HEK293 cells, as measured with a FRET-based EPAC sensor, albeit displaying 6- to 12.5-fold lower potencies (Figure 2A, Table 1). All four AZDye-labeled CXCL12 analogs induced β-arrestin2 recruitment to ACKR3 and are expected to act as a full agonist, however, their 5- to 15-fold lower potencies compared to non-labeled CXCL12 did not allow us to obtain full response curves within the tested concentration range, which was limited due to the high cost of a small amount of AZDye-tagged CXCL12 (Figure 2B, Table 1).

### 2.2. Fluorescent AZDye-Labeled CXCL12 Analogs Display Comparable Binding Affinities for ACKR3 or CXCR4

**Figure 3:**
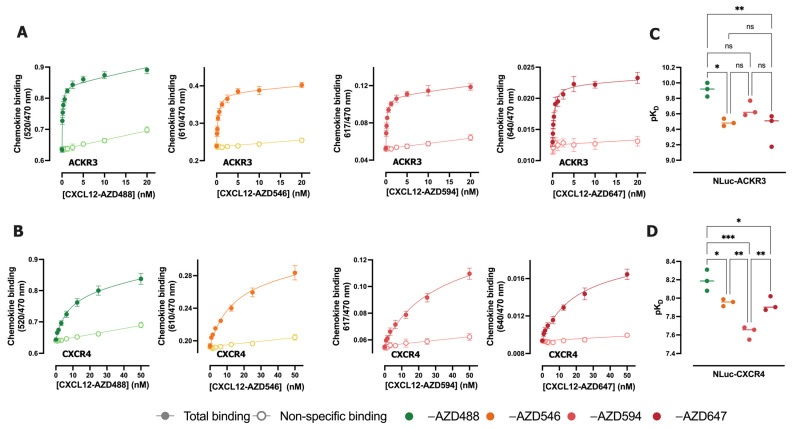
Characterization of binding properties of fluorescently labeled CXCL12 with AZDyes. (**A**,**B**) Saturation binding for increasing concentrations of CXCL12-AZDxxx to HEK293T membranes transiently expressing NLuc-ACKR3 (A) or NLuc-CXCR4 (B) in the presence or absence of 10 µM of VUF16545 or 10 µM of IT1t to determine non-specific binding, respectively. Data were fitted using the one-site total and non-specific binding model. (**C**,**D**) pK_D_ values for four CXCL12-AZDxxx. The statistical difference was tested by one-way ANOVA followed by Tukey’s multiple comparisons test and is shown as * (* *p* < 0.05, ** *p* < 0.01, *** *p* < 0.001). For the ones not indicated with *, the difference was not significant (ns). Data are shown as the mean ± SD of at least three independent experiments.

The binding of CXCL12 tagged with four different fluorescent dyes to HEK293T membranes expressing NLuc-ACKR3 or NLuc-CXCR4 was measured by NanoBRET. The highest BRET ratio values for total binding were detected for CXCL12-AZD488 (from 0.6 to 0.9) and the lowest for CXCL12-AZD647 (from 0.008 to 0.016), correlating with the overlap of the emission spectrum of the NLuc donor and the excitation spectra of the fluorescent acceptors (-AZD488 showing the highest overlap, -AZD647 showing the lowest) (Figure 3A,B). The largest assay window was observed for CXCL12-AZD594 on both ACKR3 and CXCR4, if calculated as the fold total binding over non-specific binding at K_D_ ligand concentration, which is equal to 50% receptor occupancy (Figure S1). The four fluorescent CXCL12 showed saturable binding to both ACKR3 and CXCR4, with relatively low levels of non-specific binding (Figure 3A,B). All AZDye-labeled CXCL12 display an around 50-fold higher affinity for ACKR3 compared to CXCR4, which is 10-fold higher than the observed affinity difference between these receptors for radiolabeled ^125^I-CXCL12 [25]. However, the K_D_ values obtained for CXCL12-AZD488 are the highest among all AZDye-tagged CXCL12 variants for both receptors. Additionally, the K_D_ values obtained for CXCL12-AZD546 and CXCL12-AZD647 are similar between each other for each chemokine receptor subtype (Table 1, Figure 3C,D).

### 2.3. Inhibition of CXCL12-AZDxxx Binding to NLuc-ACKR3 or NLuc-CXCR4 by Various Ligands

**Figure 4:**
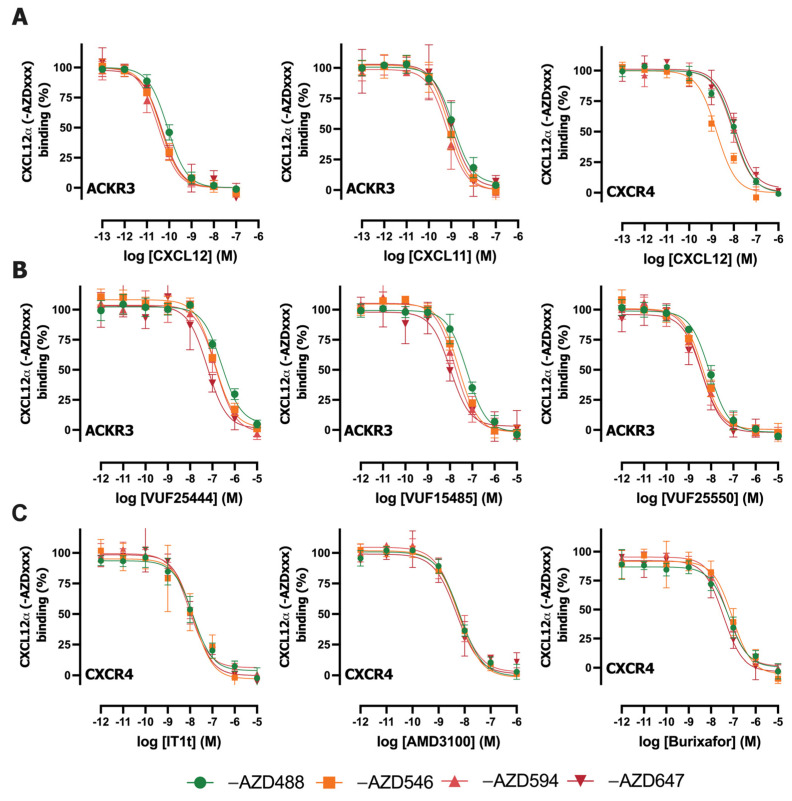
Binding inhibition of fluorescently labeled CXCL12 with AZDyes to NLuc-ACKR3 or NLuc-CXCR4 using unlabeled ACKR3 or CXCR4 ligands. (**A**) Concentration-dependent inhibition of CXCL12-AZDxxx by unlabeled the chemokines CXCL12 and CXCL11. (**B**) Concentration-dependent inhibition of CXCL12-AZDxxx to NLuc-ACKR3 by unlabeled ACKR3 small molecules. (**C**) Concentration-dependent inhibition of CXCL12-AZDxxx to NLuc-CXCR4 by unlabeled CXCR4 small molecules. Data are normalized as a percentage of the top and bottom plateau of AZDye-labeled CXCL12 binding and are shown as the mean ± SD of at least three independent experiments.

Next, the binding of the four fluorescently labeled CXCL12 to NLuc-ACKR3 and NLuc-CXCR4 was evaluated in competition with increasing concentrations of unlabeled chemokines and small-molecule ligands for these receptors (Figure 4). The pK_i_ values of the unlabeled chemokines CXCL11 and CXCL12 for ACKR3 and/or CXCR4 were similar when tested in competition with the four different AZDye-labeled CXCL12 probes (Table 2). A 6-fold higher K_i_ value was observed for unlabeled CXCL12 in competition with NLuc-CXCR4 compared to CXCL12-AZD546 and CXCL12-AZD594. (Figure 3A, Table 2). Unlabeled CXCL12 has a 6- to 18-fold and 2- to 8-fold higher affinity for Nluc-ACKR3 and Nluc-CXCR4, respectively, compared to its four AZDye-labeled analogs (Table 2).

The panel of small molecules tested in competition binding with AZDye-CXCL12 to NLuc-ACKR3 or NLuc-CXCR4 includes ACKR3 agonists VUF25444 [26] and VUF15485 [22], ACKR3 antagonist VUF25550 [19], CXCR4 antagonist IT1t [27], FDA-approved Plerixafor/AMD3100 [28] and burixafor (TG-0054) [20]. Small molecule VUF25444 yielded comparable pK_i_ values for ACKR3 when tested in competition with CXCL12-AZD488, CXCL12-AZD546, and CXCL12-AZD594, whereas a slightly higher affinity (2.5-fold) was observed when tested versus CXCL12-AZD647 (Figure 3B, Table 2). Additionally, the obtained affinity values were in line with the literature [29]. The compound VUF15485 showed comparable pK_i_ values for ACKR3 when tested in competition with CXCL12-AZD546, CXCL12-AZD594, CXCL12-AZD647. The obtained affinity values were in line with the literature [22], although a lower affinity (5-fold) was observed when tested in competition with CXCL12-AZD488 (Figure 3B, Table 2). The small molecule ACKR3 antagonist VUF25550 and CXCR4 antagonists IT1t, AMD3100, and burixafor exhibited comparable pK_i_ values for ACKR3 and CXCR4, respectively, when tested against the four AZDye-labeled CXCL12 analogs (Figure 3B, Table 2). The obtained affinity values for VUF25550 are 3- to 5-fold lower compared to its previously reported K_i_ for SNAP-tagged ACKR3 in competition with CXCL12-AF647 [19]. Our obtained K_i_ values of IT1t and AMD3100 for CXCR4 were approximately 1.5- and 30-fold higher, respectively, than previously observed in a NanoBRET-based CXCR4 binding assay versus CXCL12-AF647 [30]. However, a K_i_ value of 10 nM has also been recently reported for AMD3100 in this NanoBRET-based binding assay using CXCL12-AF647 [31], which is only 3-fold lower than observed for the four AZDye-labeled CXCL12 analogs in this study. For burixafor, an IC_50_ value of 10 nM in a CXCL12 competition binding assay on human CXCR4 was obtained [32]; this is likely in the same order range as our observed K_i_ value of 25 nM. However, considerably different K_i_ values were previously reported for AMD3100 (K_i_ = 221 ± 40 nM), IT1t (K_i_ = 0.6 ± 0.4 nM), and burixafor (K_i_ = 0.1 ± 0.02) for CXCR4 when measured in competition with a fluorescently labeled monoclonal antibody (APC-12G5) [33]. These variations in binding affinity values for unlabeled ligands in competition binding assays on chemokine receptors might be related to various probes that interact (slightly) differently with the receptor, as previously reported [34].

### 2.4. Multiplexed NanoBRET-Based Binding Assay to Simultaneously Detect Ligand Binding to Two Different Chemokine Receptors in the Same Sample

**Figure 5:**
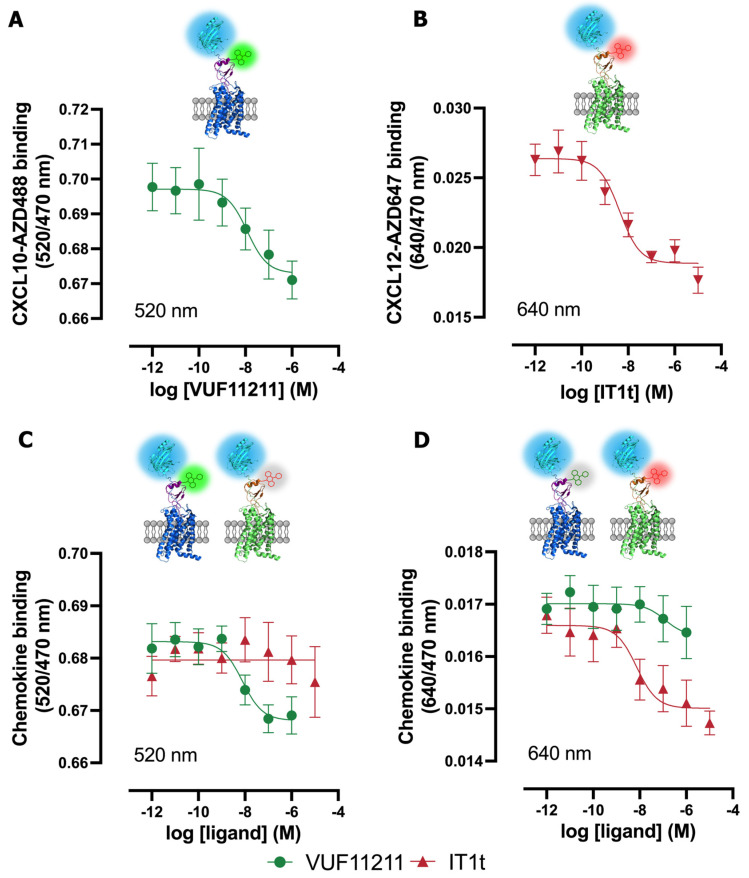
Multiplexed NanoBRET-based assay for CXCR3 and CXCR4. (**A**) Concentration-dependent binding inhibition of CXCL10-AZD488 to NLuc-CXCR3 or (**B**) CXCL12-AZD647 to NLuc-CXCR4 by unlabeled ligands. (**C**,**D**) Multiplexed NanoBRET assay for NLuc-CXCR3 and NLuc-CXCR4. Concentration-dependent inhibition of CXCL10-AZD488 (**C**) or CXCL12-AZD647 (**D**) to NLuc-tagged receptors by unlabeled small molecules. Data are plotted as BRET ratio and are shown as the mean ± SD of at least three independent experiments.

To potentially increase screening efficiency, the use of two specific chemokines with different fluorescent tags was explored in a multiplex NanoBRET assay to allow the simultaneous detection of unlabeled ligand binding to their corresponding chemokine receptors. To this end, the binding of two chemokines that are labeled with AZD488 or AZD647 to mixed (1:1 ratio) membranes expressing their cognate receptors was measured using NanoBRET by taking advantage of their separated acceptor emission spectra (Table 1, Figure 1). Since both ACKR3 and CXCR4 share CXCL12 binding, the related chemokine receptor CXCR3 was chosen in combination with CXCR4 for the multiplex NanoBRET detection of their specific interaction with CXCL10-AZD488 and CXCL12-AZD647, respectively. The small-molecule CXCR3 inverse agonist VUF11211 yielded a pK_i_ value of 8.8 in competition with CXCL10-AZD488 on NLuc-CXCR3-expressing membranes (Figure 5A), which is 1.5-fold lower or 10-fold higher than the K_i_ retrieved from competition binding with [^3^H]-VUF11211 or [^125^I]-CXCL11, respectively [23]. A similar pK_i_ was observed for VUF11211 when measured at 520 nm in the multiplex format using the mix of NLuc-CXCR3- and NLuc-CXCR4-expressing membranes in the presence of both CXCL10-AZD488 and CXCL12-AZD647 (Figure 5C), but no clear competition binding curve was detected when measured at 640 nm (Figure 5D, Table 3). The small CXCR4 antagonist IT1t yielded a pK_i_ value of 8.6 in competition with CXCL12-AZD647 on Nluc-CXCR4-expressing membranes (Figure 5A), which is 4-fold lower than the K_i_ reported in the literature [33]. A comparable pK_i_ was observed for IT1t when measured at 640 nm in the multiplex format using the mix of NLuc-CXCR3- and NLuc-CXCR4-expressing membranes in the presence of both CXCL10-AZD488 and CXCL12-AZD647 (Figure 5D), but no clear competition binding curve was detected when measured at 520 nm (Figure 5C, Table 3).

## 3. Discussion

The combination of fluorescent ligands with different emission/excitation properties and NLuc-fused GPCRs facilitates the development of NanoBRET-based binding assays. Various fluorescent tools have been employed in the last few years to monitor the interaction of ligands with chemokine receptors (such as ACKR3, CXCR4), including small molecules, nanobodies, and chemokines. In this study, CXCL12 analogs labeled with different AZDyes that cover the visible light spectrum between 490 nm and 650 nm were investigated.

Chemokines are typically tagged at their C-terminal end because the N-terminal part is crucial for the binding and activation of the cognate chemokine receptor. Even alterations in a few amino acids (truncations or substitutions) in the N-terminal part of CXCL12 can significantly affect its binding or potency towards both ACKR3 and CXCR4 [35,36,37,38]. However, this study demonstrates that tagging at the C-terminal end of CXCL12 also affects the binding affinity and potency of the CXCL12 analogs for ACKR3 and CXCR4, which is consistent the with existing literature [39,40].

As demonstrated in this study, NLuc emission can support resonance energy transfer to fluorescently tagged acceptors from a wide range of light wavelengths. NanoBRET between NLuc-ACKR3, NLuc-CXCR4 and four labeled CXCL12 analogs (CXCL12-AZD488, -AZD546, -AZD594, -AZD647) was detected. Additionally, the binding of all AZDye-labeled CXCL12s to NLuc receptors was inhibited by pharmacologically relevant unlabeled chemokines and small molecules. Knowing that fluorescently labeled chemokines covering a wide range of the light spectrum can be used as acceptors in NanoBRET provided the opportunity to conduct a multiplex NanoBRET binding assay for two chemokine receptors simultaneously for the first time. So far, multiplex-based assays have been applied to immunoassay-based protein–protein interactions [41], high-throughput GPCR antibody discovery [42,43], and the multiplexing of functional assays for GPCRs [44,45,46,47]. However, to the best of our knowledge, multiplex-based assays have not been used in the context of monitoring ligand–receptor-binding assays.

Since both ACKR3 and CXCR4 share CXCL12 binding, the related chemokine receptor CXCR3 was chosen, in combination with CXCR4, for the multiplex NanoBRET detection of their specific interaction with CXCL10-AZD488 and CXCL12-AZD647, respectively. The NanoBRET binding assay for CXCL10-AZD488 binding to Nluc-CXCR3 was successfully set up and was not previously reported in the literature. Then, the simultaneous measurement of the binding ability of unlabeled small molecules towards CXCR3 and CXCR4 using NanoBRET methods was successfully performed.

There are some points of improvement and considerations to address in the presented multiplex NanoBRET binding assay. The first point to note is that two different batches of membranes, either expressing NLuc-CXCR3 or NLuc-CXCR4, were used. For the multiplex assay in this study, membranes co-expressing both receptors were not chosen due to the confirmed formation of heteromers, which could potentially influence the multiplex data/readout [48]. Notably, when CXCR3 and CXCR4 were co-expressed, some displacement of [^125^I]-CXCL10 (specific to CXCR3) was observed by CXCL12 (a CXCR4-specific chemokine). Similar effects were noted in competition binding studies with [^125^I]-CXCL12 and unlabeled CXCL10 [48]. In both cases, these effects were not detected when CXCR3 or CXCR4 were expressed alone [48].

While using membranes co-expressing a few GPCRs could be considered for setting up a multiplex NanoBRET binding assay, it is important to determine if those receptors interact with each other. This assessment is necessary to ensure the validity of the assay results. If confirmed, this approach could potentially streamline the methods and scale up the assay in terms of the number of targets tested in one experiment.

Additionally, another important consideration when setting up multiplex NanoBRET experiments is the selection of fluorescent ligands with well-separated emission spectra. It is important to carefully choose the wavelength and bandwidth for measuring the emission of the acceptors to avoid potential bleed-through.

Furthermore, another limitation of multiplexing with the chemokine receptor family and labeled chemokines is the fact that many chemokine receptors share the same chemokine ligands. Consequently, the use of specific fluorescent chemokines for measuring binding to multiple targets can pose challenges. One solution would be to use receptor-specific fluorescently labeled small molecules or specific labeled nanobodies, which have also been employed in NanoBRET studies [7,8,30].

Another potential improvement would be to attempt multiplex assays in intact living cells to study more accurate chemokine–receptor binding, as the presence of other factors such as glycosaminoglycans (GAGs) is also involved in chemokine binding [49].

In conclusion, multiplexing the NanoBRET-based competition binding assay is a promising tool for testing unlabeled (small) molecules against multiple GPCRs simultaneously. Further optimization could potentially lead to the scaling up of the multiplex assay for high-throughput applications.

## 4. Materials and Methods

### 4.1. Materials

HBSS (with Ca^2+^ and Mg^2+^), Dulbecco’s Modified Eagle’s Medium (DMEM; high glucose), 0.05% trypsin solution and penicillin/streptomycin solution, and enzyme-free cell dissociation buffer were purchased from ThermoFisher Scientific (Waltham, MA, USA). Fetal bovine serum (FBS) was obtained from Bodinco (Alkmaar, the Netherlands). Linear 25 kDa polyethylenimine (PEI) was purchased from Polysciences (Warrington, PA, USA). Bovine serum albumin (BSA) was obtained from Melford (Ipswich, UK). Furimazine (N1130) was purchased from Promega (Madison, WI, USA). The 96-well white and black cell culture plates used were obtained from Greiner Bio-one (Kremsmünster, Austria). White low-volume 384-well plates were bought from Corning (Corning, NY, USA). Human recombinant CXCL12, CXCL10, and the fluorescently labeled chemokines CXCL12-AZD488, CXCL12-AZD546, CXCL12-AZD594, CXCL12-AZD647, and CXCL10-AZD488 were purchased from Protein Foundry (Milwaukee, WI, USA). IT1t (Bio-Techne Ireland Limited, Dublin, Ireland), AMD3100 and Burixafor were purchased from MedChemExpress (Princeton, NJ, USA), and VUF25444, VUF15485, VUF25550 and VUF16545 were synthetized in-house.

### 4.2. Constructs

The HA-tagged ACKR3 was C-terminally fused to SmBit by a TSSGSSGGGGSGGGGSS linker and subcloned in the expression plasmid pcDEF3, as previously described [50]. The NLucACKR3 and NLucCXCR4 constructs, in which NLuc is preceded by the 5HT3a signal peptide and fused via a Gly–Ser linker (BamHI restriction site) to ACKR3 or CXCR4 in pcDNA3.1, were kindly provided by Dr. Hill (Nottingham University, Nottingham, United Kingdom), whereas LgBit-β-arrestin2 was a kind gift from Dr. Seong (Korea University, Seoul, Republic of Korea). [5,51]

NLuc-CXCR3 was generated by replacing the startcodon with a BamHI restriction site using PCR and subcloning this PCR fragment into NLuc-ACKR3/pcDNA3.1 using BamHI and XbaI restriction enzymes. All generated constructs were verified by DNA sequencing.

### 4.3. Cell Culture

Human embryonic kidney 293T cells (ATTC, CRL-1573) were cultured in DMEM supplemented with 10% FBS and penicillin (100 mg/mL) and streptomycin (50 mg/mL) at 37 °C with 5% CO_2_.

Human embryonic kidney 293 cells stably co-expressing CXCR4 and the FRET-based EPAC sensor with mCerulean and mCitrine as a FRET pair were kindly provided by Dr. M. Zimmermann (Interax Biotech, Zurich, Switzerland), and were cultured in DMEM supplemented with 10% FBS and penicillin (100 mg/mL), streptomycin (50 mg/mL), zeocin (0.06 mg/mL) and G418 (0.6 mg/mL) at 37 °C with 5% CO_2_.

### 4.4. β-arrestin2 Recruitment (NanoBit)

HEK293T cells (3 × 10^6^/well) were plated in 10 cm^2^ culture dishes and, after 24 h, co-transfected with 0.4 µg of ACKR3-SmBit, 0.6 µg of human LgBit-β-arrestin2, 3 µg of pcDEF3 and 12 µg of PEI. After 24 h of transfection, the cells were collected using enzyme-free cell dissociation buffer and incubated for 10 min at 37 °C with NanoGlo substrate (1000-fold final dilution from stock). Next, the cells were dispensed into white 96-well plates (1 × 10^5^ cells/well) and incubated with various concentrations of unlabeled CXCL12 or fluorescently labeled CXCL12 at 37 °C for 15 min. The luminescence at a 470 nm wavelength with an 80 nm bandwidth was measured using the CLARIOstar Plus (BMG Labtech, Ortenberg, Germany).

### 4.5. Intracellular cAMP Measurement by EPAC Sensor

HEK293 cells stably expressing CXCR4 and a FRET-based EPAC sensor were seeded into a 96-well black plate (0.5 × 10^6^/well). After 24 h, the culture medium was replaced with HBSS with 0.05% BSA and the cells were pre-incubated at 37 °C for 10 min with 100 nM of isoprenaline to increase the endogenous cAMP level, followed by 15 min of stimulation with increasing concentrations of unlabeled CXCL12 and fluorescently labeled CXCL12. Fluorescence was measured using the following parameters: excitation at 430–15 nm, and emission at 530–20 and 480–20 nm using the CLARIOstar Plus (BMG Labtech, Ortenberg, Germany). The FRET ratio was calculated by dividing the signal of the acceptor at 530 nm by the donor signal at 430 nm.

### 4.6. NLuc-ACKR3, NLuc-CXCR4, NLuc-CXCR3 HEK293T Membrane Preparation

HEK293T cells (2 × 10^6^) were seeded in 10 cm^2^ culture dishes. After 24 h, the cells were transfected with 0.25 µg of plasmid DNA encoding human NLuc-ACKR3, NLuc-CXCR4 or NLuc-CXCR3, and 4.75 µg of pcDEF3 plasmid DNA using 30 µg of linear PEI. After two days, the cells were collected in PBS and centrifuged at 2700 rpm at 4 °C. Next, the cell pellet was resuspended in ice-cold membrane buffer (15 mM of Tris, 0.3 mM of EDTA, 2 mM of MgCl_2_, pH 7.4 at 4 °C) and homogenized by plunging the pestle using 10 strokes at 1100 rpm. The cell homogenates were exposed to two freeze and thaw cycles using liquid nitrogen and were centrifuged at 25,000 rpm. The pellet was resuspended in Tris-sucrose buffer (20 mM of Tris, 250 mM of Sucrose, pH 7.4 at 4 °C). Finally, cell membranes were homogenized using a 23-gauge needle, followed by snap-freezing with liquid nitrogen and then stored at −80 °C.

### 4.7. NanoBRET-Based Binding Assay

For the saturation binding assay, increasing concentrations of fluorescently labeled CXCL12 (CXCL12-AZD488, -546, -594 or -647) were incubated with HEK293T membranes expressing NLuc-ACKR3 or NLuc-CXCR4 in HBSS supplemented with 0.2% BSA in a white low-volume 384-well assay plate, in the absence or presence of 10 μM of VUF16545 or IT1t, respectively, to determine non-specific binding. For competition binding assays, HEK293T membranes expressing NLuc-ACKR3 or NLuc-CXCR4 were incubated with 0.3 or 10 nM of the four fluorescently labeled CXCL12, respectively, with increasing concentrations of unlabeled ligand. After 1 h of incubation at room temperature, furimazine substrate was added (310-fold final dilution from stock). The luminescence was measured at a 470 nm wavelength with an 80 nm bandwidth and separately for the wavelengths of 520–20 nm, 570–80, 617–100, 640–80 using the CLARIOstar Plus (BMG Labtech, Ortenberg, Germany) with a dual emission filter. The ratio of luminescence at the acceptor channel to the donor channel is a measure for the relative binding of CXCL12-AZDxxx to the NLuc-tagged receptor.

### 4.8. Multiplex NanoBRET-Based Binding Assay to CXCR4 and CXCR3

The binding of 10 nM of fluorescently labeled CXCL12-AZD647 and/or 10 nM of fluorescently labeled CXC10-AZD488 to mixed (1:1) or individual HEK293T membranes expressing either NLuc-CXCR4 or NLuc-CXCR3, respectively, in the presence of increasing concentrations of an unlabeled ligand was measured in HEPES binding buffer (25 mM of HEPES, 1 mM of MgCl_2_, pH 7.4) supplemented with 0.2% BSA in a white low-volume 384-well assay plate. NanoBRET was measured after 1 h of incubation at room temperature, as described above.

### 4.9. Data Analysis

All experiments were analyzed using Graphpad Prism 9.0. For binding experiments, pIC_50_ values were obtained using a one-site pIC_50_ model using global fitting with shared non-specific binding values.

For saturation binding experiments, the K_D_ was obtained using one-site total and non-specific binding. For binding experiments, pIC_50_ values were obtained using a one-site pIC_50_ model. The equilibrium dissociation constants (Ki) of unlabeled ligands were subsequently calculated using the Cheng−Prusoff equation.

(1) $$K_{1}=\frac{{IC}_{50}}{1+\frac{L}{K_{D}}}$$

where [L] is the concentration of the labeled ligand and KD is the equilibrium dissociation constant of the labeled ligand.

## Figures and Tables

**Table 1:** Pharmacological characterization of fluorescently labeled CXCL12 with AZDyes on ACKR3 and CXCR4.

Ligand	Excitation Maximum (nm)	Emission Maximum (nm)	cAMP by CXCR4 (pEC_50_)	β-arrestin2 Recruitment to ACKR3 (pEC_50_)	pK_D_ for CXCR4 ^c^	pK_D_ for ACKR3 ^c^
CXCL12	-	-	9.7 ± 0.2 ^a^	8.0 ± 0.1 ^a^	-	-
-AZD488	494	517	8.8 ± 0.3	7.3 ± 0.2	8.2 ± 0.1	9.9 ± 0.1
-AZD546	554	570	8.6 ± 0.3	6.8 ± 0.3	8.0 ± 0.0	9.5 ± 0.0
-AZD594	590	617	8.9 ± 0.2	6.9 ± 0.2	7.6 ± 0.1	9.7 ± 0.1
-AZD647	649	671	8.8 ± 0.2	7.5 ± 0.1 ^b^	7.9 ± 0.1	9.4 ± 0.2

Data are shown as the mean ± SD of at least three independent experiments. Statistical differences in pEC_50_ were determined using one-way ANOVA with Tukey’s multiple comparison test and are indicated as follows: ^a^ Statistical difference (*p* < 0.05) between pEC_50_ values of CXCL12 and the four CXCL12-AZDxxx; ^b^ Statistical difference (*p* < 0.05) between pEC_50_ values of CXCL12-AZD647 versus CXCL12-AZD546 and CXCL12-AZD594. ^c^ Statistical analysis of pK_D_ values is shown in Figure 3C,D.

**Table 2:** Binding affinities (pK_i_) of reference ligands for NLuc-ACKR3 and Nluc-CXCR4 derived from competition binding with the four AZDye-labeled CXCL12 probes.

Receptor	Ligand	-AZD488	-AZD546	-AZD594	-AZD647
ACKR3	CXCL12	10.6 ± 0.1	10.7 ± 0.1	10.8 ± 0.2	10.5 ± 0.1
	CXCL11	9.4 ± 0.3	9.4 ± 0.3	9.5 ± 0.3	9.2 ± 0.3
	VUF25444	7.1 ± 0.1	7.2 ± 0.0	7.2 ± 0.2	7.5 ± 0.2 ^a^
	VUF15485	7.9 ± 0.1 ^b^	7.9 ± 0.1	8.1 ± 0.2	8.2 ± 0.1
	VUF25550	8.6 ± 0.1	8.7 ± 0.1	8.8 ± 0.3	8.7 ± 0.4
CXCR4	CXCL12	8.4 ± 0.1	9.0 ± 0.1 ^c^	8.2 ± 0.2	8.4 ± 0.5
	IT1t	8.2 ± 0.2	8.2 ± 0.4	8.2 ± 0.0	8.1 ± 0.2
	AMD3100	8.6 ± 0.1	8.5 ± 0.0	8.5 ± 0.2	8.6 ± 0.2
	Burixafor	7.6 ± 0.2	7.3 ± 0.2	7.5 ± 0.1	7.7 ± 0.1

pK_i_ values were calculated using the Cheng−Prusoff equation. Data are shown as mean ± SD of at least three independent experiments. Almost all obtained pK_i_ values are non-significantly different, except for the ones indicated with ^a,b,c^. ^a^ The statistical difference (<0.05) between the pK_i_ values of ligands retrieved from competition binding versus CXCL12-AZD488 and CXCL12-AZD647 probes was tested by one-way ANOVA followed by Tukey’s multiple comparisons test. ^b^ The statistical difference (*p* < 0.05) between the pK_i_ values of ligands retrieved from competition binding versus CXCL12-AZD488 and CXCL12-AZD647 or CXCL12-AZD594 probes was tested by one-way ANOVA followed by Tukey’s multiple comparisons test. ^c^ The statistical difference (*p* < 0.05) between the pK_i_ values of ligands retrieved from competition binding versus CXCL12-AZD546 and CXCL12-AZD594 probes was tested by one-way ANOVA followed by Tukey’s multiple comparisons test.

**Table 3:** Binding affinities (pK_i_) for CXCR3 and CXCR4 ligands.

Ligand	CXCR3 Only at 520 nm	CXCR4 Only at 640 nm	Multiplex at 520 nm	Multiplex at 640 nm
VUF11211	8.8 ± 0.2	ND	8.8 ± 0.1	ND
IT1t	ND	8.6 ± 0.3	ND	8.4 ± 0.1

pK_i_ values were calculated using the Cheng–Prusoff equation with 10 nM of CXCL10-AZD488 (pK_D_ = 8.6 ± 0.2) and CXCL12-AZD647 (pK_D_ = 7.9 ± 0.1). Data are shown as mean ± SD of at least three independent experiments. ND = value could not be determined. Non-statistical differences (*p* > 0.05) between pK_i_ values from the non-multiplex vs. multiplex assay were confirmed by a t-test with Welch’s correction.

## Data Availability

The original contributions presented in the study are included in the article/Supplementary Material, further inquiries can be directed to the corresponding author.

## Supplementary Materials

The following supporting information can be downloaded at: https://www.mdpi.com/article/10.3390/ijms25095018/s1.
