# Supplementary material for: Multiplex Detection of Fluorescent Chemokine Binding to CXC Chemokine Receptors by NanoBRET
Source: Int J Mol Sci. 2024 May 4;25(9):5018. doi: 10.3390/ijms25095018 (PMC11084278; doi:10.3390/ijms25095018)
Supplement: Supplementary file 1 [file ijms-25-05018-s001.zip › ijms-2962474-supplementary.pdf]

Multiplex Detection of Fluorescent Chemokine Binding to CXC Chemokine Receptors by NanoBRET

Justyna M. Adamska , Spyridon Leftheriotis, Reggie Bosma, Henry F. Vischer and Rob Leurs *

Amsterdam Institute of Molecular and Life Sciences, Division of Medicinal Chemistry, Faculty of Science, Vrije Universiteit Amsterdam, De Boelelaan 1083, 1081 HV Amsterdam, The Netherlands; spyleftheriotis@gmail.com (S.L.); h.f.vischer@vu.nl (H.F.V.)

* Correspondence: r.leurs@vu.nl

Supplementary Materials:

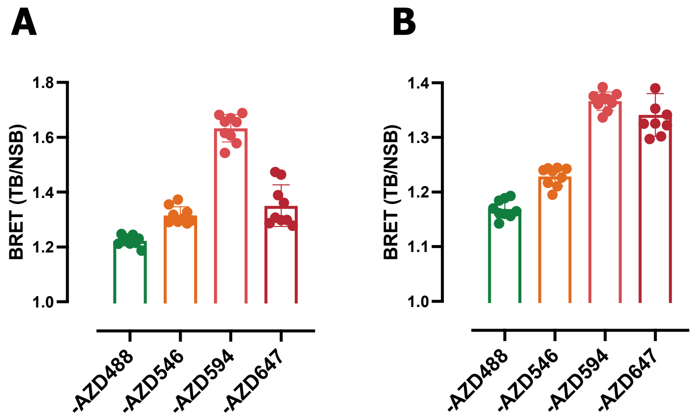

Figure S1. Fold total binding (TB) over non-specific (NSB) CXCL12-AZDxxx binding at K_D concentration for Nluc-ACKR3 (0.3 nM) (**A**) or NLuc-CXCR4 (12.5 nM) (**B**), resulting in 50% receptor occupancy. Data are shown as the BRET-ratio (BRET signal at acceptor of certain CXCL12-AZD divided by NanoLuciferase signal at 470 nm). Data are shown as the mean ± SD of at least 3 independent experiments.

Table S1. List and structures of used small molecules.

Small molecule	Structure	Mode of action
VUF16545	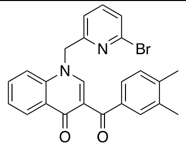	Agonist [49]
VUF25444	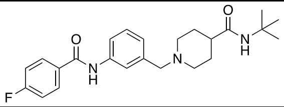	Agonist [26])
VUF15485	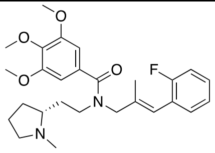	Agonist [22]
VUF25550	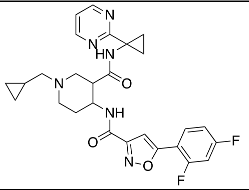	Antagonist [19]

VUF11211	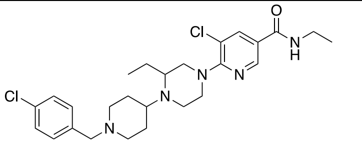	Inverse agonist [50], [51]
IT1t	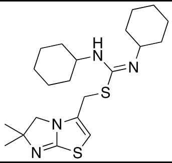	Antagonist [27]
AMD3100	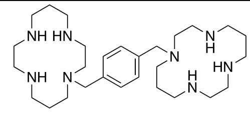	Antagonist [52]
Burixafor	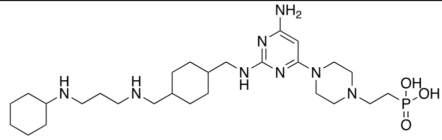	Antagonist [20]
